# Intracoronary Hydatid Cyst Resulted in Coronary Artery Disease in a Young Patient

**DOI:** 10.21470/1678-9741-2018-0033

**Published:** 2019

**Authors:** Unsal Vural, Ahmet Arif Aglar, İlyas Kayacioglu

**Affiliations:** 1 Departament Cardiovascular Surgery, Doktor Siyami Ersek Gogus Kalp ve Damar Cerrahisi Egitim ve Arastirma Hastanesi Ringgold Standard Institution, Istanbul, Turkey.

**Keywords:** Albendazole, Parasitology, Echinococcosis, Heart Disease

## Abstract

Among all cystic echinococcosis cases, only 0.5%-2% exhibit a cardiac involvement. Only 10% of these become symptomatic. Considering the long time interval between the start of infestation and symptoms to occur, it is hard to diagnose cystic echinococcosis. When detected, even if it is asymptomatic, intramyocardial hydatid cyst requires surgical intervention due to risks of spontaneous rupture and anaphylaxis. In literature, no case of hydatid cyst located in the coronary arterial wall has been reported. Twenty-two-year-old male patient with previous history of pulmonary cystic echinococcosis was referred to us with typical symptoms of coronary artery disease. Coronary cineangiography revealed proximal left diagonal artery (LAD) occlusion. Pre-operative transthoracic echocardiography of the patient planned to undergo coronary artery bypass grafting unveiled an intracoronary calcified cystic mass. In operation, the calcified cystic mass with well-defined borders and size of 2x2 cm located within wall of proximal segment of the LAD artery was excised and double bypass with left internal thoracic artery (LITA) and great saphenous vein grafts to the LAD and first diagonal arteries, respectively, was done. Pathological analysis of the mass revealed it to be an inactive calcified hydatid cyst. Echinococcal IgG-ELISA test was positive. 12-week oral albendazole treatment (2x400 mg/day) was launched postoperatively and the patient was discharged on 7^th^ postoperative day.

**Table:** 

Abbreviations, acronyms & symbols	
CT	= Computed tomography
ECG	= Echocardiography
LAD	= Left diagonal artery
LITA	= Left internal thoracic artery

## INTRODUCTION

The liver, followed by the lung, is the most common site of involvement for the hydatid cyst. The incidence of concomitant liver and lung hydatidosis varies from 5.8 to 13.3%^[1]^. These cysts grow at a rate of roughly 1 cm per year, and become symptomatic within 5 to 10 years^[2]^. Parasitic larvae migrate to heart mainly via coronary circulation. Frequency of cardiac hydatidosis vary from 0.5 to 2%^[3]^. The embryo reaches full growth at 1 to 5 years after being lodged in the heart. The myocardial reaction to the cyst creates an adventitial pericyst layer^[4]^. We aimed to present a rarely-seen location and thus complication, namely the coronary artery disease, of cardiac hydatidosis to attention.

## CASE REPORT

**Fig. 1:**
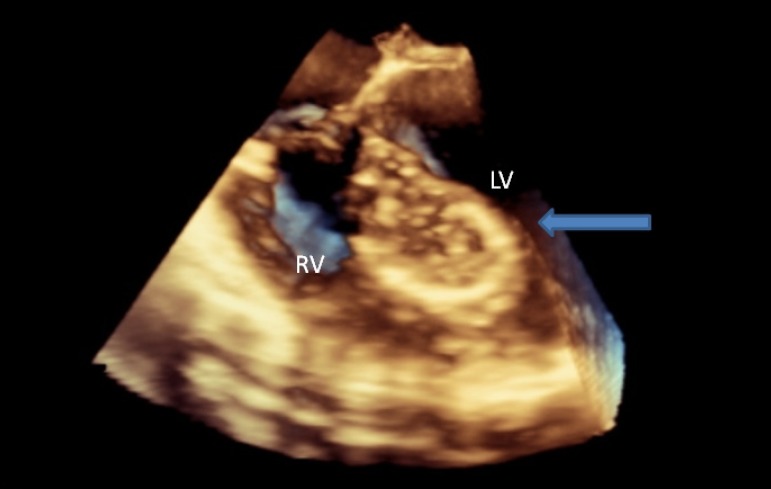
Echocardiographic view of the hydatid cyst. LV=left ventricle; RV=right ventricle

**Fig. 2:**
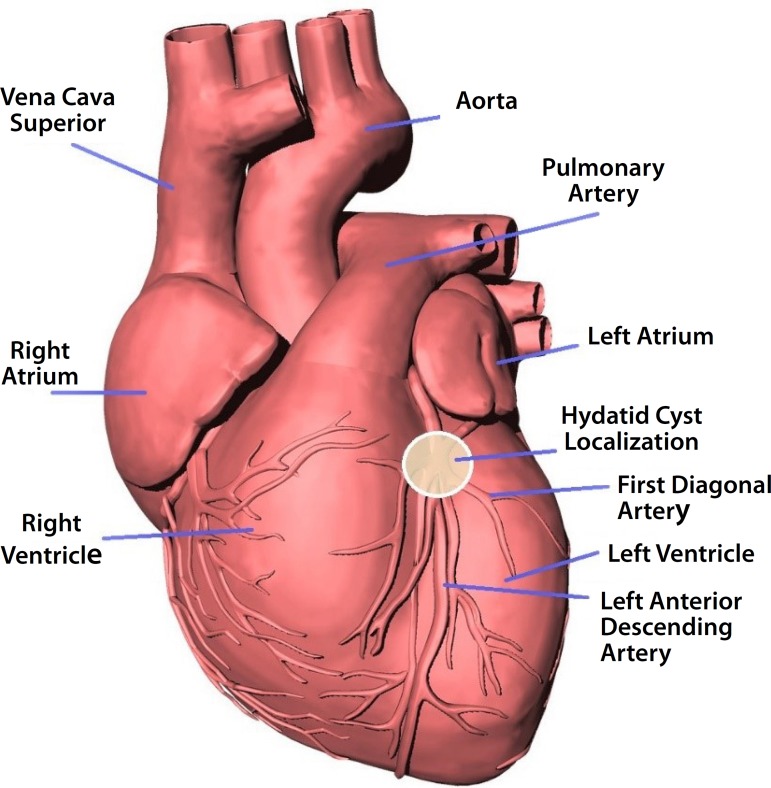
Localization of the intracoronary hydatid cyst.

**Fig. 3:**
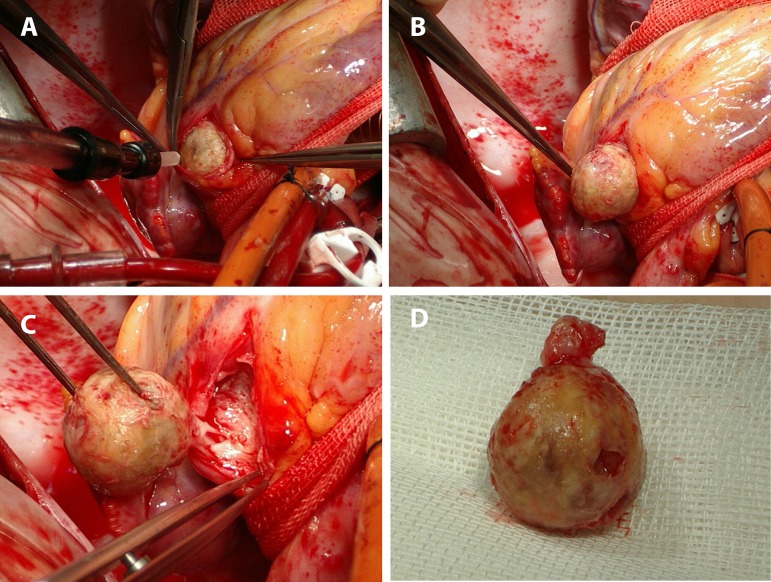
A: Perioperative view of the intracoronary hydatid cyst prior to enucleation (Size: 2x2 cm). B: Perioperative view of the intracoronary hydatid cyst, distally liberated but proximal attachment is intact. C: Perioperative view of the intracoronary hydatid cyst with proximal attachment (inferiorly located) intact. D: Enucleated intracoronary hydatid cyst, proximal attachment placed superiorly (size: 2x2 cm).

A 22-year old male patient was referred to our clinic with exertional angina. His history revealed pulmonary hydatidosis treated through cystectomy and capitonnage followed by 12-week oral albendazole treatment (400 mg/twice a day) five years ago. Chest X-ray and thoracic computed tomography (CT) exposed only few scattered calcifications within pulmonary parenchyma (Figure 1). Cineangiography of the patient with ST segment depression in exercise test revealed that the left diagonal artery (LAD) and 1^st^ diagonal arteries were proximally occluded (Figure 2). In transthoracic echocardiography, a cystic mass of 2x2 cm with well-defined borders was detected on the left ventricular anterior wall (Figure 3). The patient was taken into operation for coronary artery bypass grafting. Operation was carried out under cardiopulmonary bypass instituted after median sternotomy. The mass, with size of 2x2 cm and regular borders, was found to be located between the left main and LAD coronary arteries (Figure 3). Since calcified, the cyst was isolated *en bloc* with the coronary artery segments it had infiltrated (Figure 3). Free ends of the coronary arteries opening into the cavity left behind after cyst excision were ligated. Capitonnage was performed after irrigation with hypertonic saline solution. Following that, LAD artery and 1^st^ diagonal artery were bypassed. Macroscopically, it was detected that the cyst contains clear colorless fluid (eau de rock). Microbiological and pathological analysis of both the cyst and its ingredients revealed findings consistent with the hydatid cyst. Echinococcal IgG-ELISA test was found to be positive (sensitivity: 94%, specificity: 99%)^[5]^. The patient received oral albendazole treatment (10 mg/kg/day; 2x400 mg/day) postoperatively for 12 weeks. During 1-year follow-up, diagnostic tests were negative and no recurrence was observed.

## DISCUSSION

Cardiac hydatidosis involves mainly the left ventricle (55-60%) due to its dominant circulatory network, followed, in decreasing incidence, by right ventricle (15%), interventricular septum (9%), left atrium (8%), pericardium (7%), pulmonary artery (6-7%), right atrium (4%), and interatrial septum (2%)^[6]^. However, to date, no coronary arterial involvement of hydatid cyst causing coronary artery occlusion has been reported.

Though coughing, palpitation and angina could be the symptoms, only 10% of the cardiac hydatidosis cases become symptomatic^[7]^. In symptomatic cases, since potential cyst rupture could end up with anaphylaxis, early surgical intervention is recommended^[8]^. Ben-Hamda et al.^[8]^ reported that hydatidosis, particularly in elderly patients, could mimic angina and myocardial infarction as a result of compression to adjacent tissues. In younger patients, symptoms may comprise rather of dyspnea, coughing, loss of weight, and fever^[9]^. Though the case we present is young, he presented symptoms typical of elders. Rupture into cardiac chambers may cause ventricular outflow tract obstruction, low cardiac output syndrome, pulmonary, cerebral or peripheral emboli and hypertension. In case of conduction system involvement, rhythm problems can occur^[8]^. Moreover, as in our case, referral with coronary artery disease related symptoms is possible, too. Q and inverted T waves seen in ECG can be helpful in diagnosis. Echocardiography is an effective tool in diagnosis of cardiac hydatidosis. CT and magnetic resonance imaging are effective, particularly in diagnosis of extracardiac hydatidosis. In the case we present, intracoronary calcified inactive cyst is thought to be originated from embolization of ruptured pulmonary hydatid cyst since his history reveals previous pulmonary involvement. It is postulated that, as a result of either previous medical treatment or spontaneous cyst inactivation, the cystic growth was impeded, and the cyst was calcified. Therefore, the cyst was easily enucleated. When findings suggestive of cardiac involvement are present, particularly in endemic areas (Mediterranean coasts, Middle East, Australia, South America), cardiac hydatidosis must be considered^[2]^.

Surgical intervention is the first-line treatment since medical therapy is insufficient in preventing the rupture of the cyst^[8]^. As in the case we present, coronary complication of pulmonary hydatidosis could be seen even 5 years after the treatment. Some surgeons proposed perioperative sterilization of the cystic location with either 2% formalin or 20% hypertonic saline solution^[2]^. The cyst in our case was easily isolated *en bloc* with coronary artery segments it infiltrated without any rupture. Free ends of the coronary arteries opening into the cavity left behind after cyst excision were ligated. Capitonnage was performed after irrigation with hypertonic saline solution. Following that, LAD artery and 1^st^ diagonal artery were bypassed.

Definitive diagnosis and assessment of cystic activity are done through cyst serology. Though echinococcal IgG-ELISA test is sensitive, the treatment strategy must not be directed by cyst serology. This is due to the fact that serology could be negative while hydatid cyst infestation is present or be positive for years while a treated and inactive cyst is present^[10]^. For this reason, considering all possibilities, oral albendazole treatment (10 mg/kg/day; 2x400 mg/day) postoperatively for 12 weeks was administered.

In young cases of hydatidosis, since clinical presentations resemble those of other conditions, without further studies it is hard to trace angina to cardiac involvement rather than pulmonary hydatidosis. Particularly in regions where hydatidosis is endemic, in differential diagnosis of young patients with coronary artery disease of unknown origin, cardiac hydatidosis must be considered. Moreover, while surgical intervention and medical therapy are planned for pulmonary hydatidosis, possibility of concomitant organ involvement must not be passed over.

**Table:** 

Authors' roles & responsibilities	
UV	Substantial contributions to the conception of the work; the analysis and interpretation of data for the work; final approval of the version to be published
AAA	Substantial contributions to the conception of the work; the analysis and interpretation of data for the work; final approval of the version to be published
IK	Substantial contributions to the conception of the work; the analysis and interpretation of data for the work; final approval of the version to be published
